# Supplementary material for: Perception of urban green space among university students in Bangladesh
Source: PLoS One. 2024 Sep 30;19(9):e0311033. doi: 10.1371/journal.pone.0311033 (PMC11441694; doi:10.1371/journal.pone.0311033)
Supplement: Attachment — Submitted filename: PloS one review.pdf [file pone.0311033.s002.pdf]

PloS One Review – Perception of urban green space among university students in Bangladesh

Abstract – intangible services?

Line 57 – 59 → need connecting sentence

Line 58 – more recent data than 2016?

63 – doesn't make sense – how does

78 – scope to include lit on green space perception

81 – really? I beg to differ!

84 – enlightening uni students? Focus/justification of study unclear

93 – a better sub-heading would be 'Determinants of urban green space use'

98 – who was surveyed?

99 – sentence fragment

102 – how does one know whether it's not the wealth that leads to happiness?

108 – urban green what?

114 – top concern from what?

116 – 'activities' is unclear.

114-130 – this seems confused – better to write thematically.

167 – unclear

182 – financial returns unclear

202 – developing nations, then big leap to students. A clearer connection needs to be made.

209 – space needed

210-211 – climate change and agricultural dependence is unclear

217 – research design approach (exploratory) needs to be supported by literature

229 – conflict of interest?

265 – why and how? A lot of unqualified assertions are being made

315 – implies only students who were interested in green spaces were surveyed

316 – tense changes

317-318 – Survey design is based on the work by Bonaiuto et al. (2003). There is no indication of what these perceptions are nor is this mentioned in the Literature Review.

423-424 – when students were surveyed re. locality, did this mean usual place of residence or while studying at the university?

673 – students are adults

681 – women manage households? How is that connected to UGS?

682 – age not a factor; rather level of education

709 – heightened feeling of UGS unclear… positive? Awareness of? Importance?

755 – quali data? Unclear – only quant used

Suggested literature for Literature Review

Cleary, A., Roiko, A., Burton, N.W., Fielding, K.S., Murray, Z. and Turrell, G., 2019. Changes in perceptions of urban green space are related to changes in psychological well-being: Cross-sectional and longitudinal study of mid-aged urban residents. *Health & place*, *59*, p.102201

Gozalo, G.R., Morillas, J.M.B. and González, D.M., 2019. Perceptions and use of urban green spaces on the basis of size. *Urban Forestry & Urban Greening*, *46*, p.126470.

Gozalo, G.R., Morillas, J.M.B., González, D.M. and Moraga, P.A., 2018. Relationships among satisfaction, noise perception, and use of urban green spaces. *Science of the total environment*, *624*, pp.438-450.

Palliwoda, J. and Priess, J.A., 2021. What do people value in urban green? Linking characteristics of urban green spaces to users' perceptions of nature benefits, disturbances, and disservices. *Ecology & Society*, *26*(1).

Ugolini, F., Massetti, L., Calaza-Martínez, P., Cariñanos, P., Dobbs, C., Ostoić, S.K., Marin, A.M., Pearlmutter, D., Saaroni, H., Šaulienė, I. and Vuletić, D., 2022. Understanding the benefits of public urban green space: How do perceptions vary between professionals and users?. *Landscape and urban planning*, *228*, p.104575.
